# Kids' Perceptions toward Children's Ward Healing Environments: A Case Study of Taiwan University Children's Hospital

**DOI:** 10.1155/2016/8184653

**Published:** 2016-10-05

**Authors:** Jeng-Chung Woo, Yi-Ling Lin

**Affiliations:** ^1^Department of Product Design, Sanming University, Sanming City, Fujian, China; ^2^Department of Arts and Plastic Design, Taipei University of Education, Taipei City, Taiwan

## Abstract

This paper summarizes the opinions of experts who participated in designing the environment of a children's hospital and reports the results of a questionnaire survey conducted among hospital users. The grounded theory method was adopted to analyze 292 concepts, 79 open codes, 25 axial codes, and 4 selective codes; in addition, confirmatory factor analysis and reliability analysis were performed to identify elements for designing a healing environment in a children's hospital, and 21 elements from 4 dimensions, namely, emotions, space design, interpersonal interaction, and pleasant surroundings, were determined. Subsequently, this study examined the perceptions of 401 children at National Taiwan University Children's Hospital. The results revealed that, regarding the children's responses to the four dimensions and their overall perception, younger children accepted the healing environment to a significantly higher degree than did older children. The sex effect was significant for the space design dimension, and it was not significant for the other dimensions.

## 1. Introduction

In the 1990s, “healing environments” became a crucial concept in designing and planning medical facilities. Healthcare operators consider their medical institutions to be healing environments, regardless of whether they can provide a definition for the term “healing environment” [1]. Currently, healing and treatment should be considered simultaneously in the establishment of a new healthcare model [2].

Wetton [3] stated that emotional design provides pleasure to viewers or users, a notion that has been widely accepted and applied in healthcare to satisfy patients' psychological needs. For example, a toy baby seal (Paro) was designed for nursing homes and hospitals in Japan and Europe [4]. Toys and games are the focus of children's lives, and they enable people to communicate with children and help doctors in establishing a relationship with children [5]. In nursing homes, elderly people who care for plants and participate in activities are physically and psychologically active and feel happy [6]. Healing design is widely accepted and involves using various types of software and hardware to heal patients.

A healing environment can be defined as a holistic environment that facilitates patient rehabilitation. In contrast to medical treatment, healing is a psychological concept of health [7]. Evidence-based design has become a theoretical concept in the creation of healing environments [8, 9]; healthcare providers prefer using evidence-based information to make decisions. Children and adolescents are often neglected in fields such as architectural and urban planning, although they are typically more sensitive to environments compared with adults [10]. Scanlon and Bauer [11] emphasized that because young children are more dynamic than adults are, more considerations should be included in healthcare for children. Therefore, developing an evidence-based method for designing a healing environment for a children's hospital is an appealing topic that warrants further research. Planning for National Taiwan University Children's Hospital (NTUCH) began in 1994, and the facility officially opened in December 2008. NTUCH was the first children's hospital in Taiwan to adopt a healing environment design. However, whether the design conforms with users' perception of a healing environment and whether it achieves the objective of such an environment must be evaluated. In addition, previous studies on hospital environments have primarily examined user satisfaction [12–14] and special tasks; however, only few studies have provided useful insights into the design-related interaction between users and their environments [15]. Therefore, the objectives of this study were twofold:

1. Identify an evidence-based method for designing and establishing a healing environment that meets the requirements of children's hospitals.
2. Determine whether the atmosphere created by the NTUCH healing environment design conforms with users' perception of a healing environment and whether it achieves the objectives of such an environment.

## 2. Literature Review

The “patient-centered” care concept poses a challenge to many healthcare service provision practices [16]. In 1978, Planetree, a nonprofit organization, was founded. Planetree emphasizes the importance of a patient-centered healing environment. The Society of Arts in Healthcare, founded in the United Kingdom in 1991, integrates art and healthcare facilities in its endeavor to provide an excellent healing environment for patients by reducing their stress, enhancing their sense of security, and improving their physical and mental health [17]. In 1993, the Center for Health Design in the United States was founded to advocate for a secure and healthy healthcare environment. This organization has facilitated the study of healing environment designs, and it launched the Pebble project to promote healing environments worldwide.

Biley [29] stated that rich and diverse visual (e.g., gardens, pictures, and colors) and auditory (e.g., relaxing music and natural sounds) environments facilitate the creation of a healing environment. The enjoyment derived from nature can distract patients from their ailments, thus alleviating their pain [30]. Ulrich [31] demonstrated that nature, green plants, and an extensive space can effectively reduce stress, alleviate pain, improve body immunity, and enhance human resilience. Improving healthcare environments can facilitate patients' recovery, enable patients to relax, and reduce the stress of healthcare staff, thereby indirectly improving doctor-patient relationships, enhancing healthcare quality, and reducing healthcare costs. The National Association of Children's Hospitals and Related Institutions (NACHRI) [32] indicated that active distraction strategies such as providing opportunities for patients to engage in art and music can reduce stress and anxiety and that natural sunlight can alleviate depression. Hence, exploring these topics related to designing a healing environment for children and adolescents is worthwhile.

Creating healing environments in children's hospitals is a current trend [33]. Psychologists have indicated that physical environments can influence treatment processes and results [34]. Moreover, environmental health is considered in healthcare engineering [35]. A physical environment can be divided into two categories: indoor and outdoor environments. Previous studies have reported that factors that influence a healing environment are safety, sound [29], color [2], artwork [36, 37], interactive art [38], lighting [39], outdoor view [40], furnishing [41], and atmosphere [42]. Moore [43] also indicated that playing outdoors positively influences the interaction between children and the society. Nature and gardens can effectively improve the emotions of children and adolescents, alleviate their pain, and produce a healing effect [18, 32]. The design features of healing environments should be able to provide users with multisensory experience as well as positive healing effects [37]. Ozcan [10] also revealed that face-to-face social interaction can enhance healing. In addition, Altimier [12] indicated that an outdoor view, natural sunlight, pastel colors, therapeutic sounds, and interaction with family members can facilitate healing; Altimier reported that these elements must be balanced with staff members' requirements in the design of essential care environments.

Evidence-based design is a theoretical concept applied for creating healing environments [8, 9]. In healing processes, actual environments are related to the well-being of patients, their family members, and healthcare staff [8, 13]; however, the perspectives of these people regarding healthcare design are often neglected during decision-making [9]. A study reported that nursing staff members' self-care was negatively correlated with their compassion fatigue and burnout [44]. Caring can strongly influence the efficiency of healing environments, such as patients' interaction with their caregivers. One of the goals of a healing environment is to restore wholeness through helpful design features that address the thoughts and emotions of patients and care providers. Moreover, Planetree advocates the concept of healthy healthcare service providers [38]. Incorporating a healing environment into healthcare also optimizes clinical care and outcomes as well as employee satisfaction and morale in addition to patient satisfaction [12].

The patient-centered concept entails enhancing patient well-being related to an aesthetic, comfortable, safe, and pleasant atmosphere; encouraging patients to interact with their family members, caregivers, and other patients; and providing a homelike environment [38, 45]. The designs of numerous hospitals have been based on experts' perspectives and have prioritized cost efficiency and clinical functionality; specifically, only the perspectives of management staff, architects, and policymakers have been considered in such designs [46]. Therefore, evidence-based healthcare design should consider hospital users' perception of a care unit [9].

As mentioned, a healing environment can be defined as a holistic environment (physical and nonphysical) that facilitates patient rehabilitation. In contrast to treatment, healing is a psychological concept of health [7]. However, children are generally neglected in architectural planning, despite them being typically more sensitive to environments than adults are [10]. Scanlon and Bauer [11] emphasized that young children are more dynamic than adults are and that various considerations should be included in children's healthcare because children's requirements differ from those of adults. Developing an evidence-based method for designing a healing environment for children's hospitals is an appealing topic that warrants further research.

## 3. Method and Design

### 3.1. Questionnaire Development

The ethics committee of the National Taiwan University Hospital approved this study. Moreover, all participants provided informed consent and agreed to participate voluntarily. The questionnaire development involved six stages:

1. To obtain background knowledge for interviews, a literature review was conducted according to the connotations of a healing environment in a children's hospital.
2. The interview topic was related to the design of a healing environment in a children's hospital. On the basis of the literature review, a questionnaire for in-depth semistructured interviews was developed (Supplementary Material A, in the Supplementary Material available online at http://dx.doi.org/10.1155/2016/8184653), and the interviewees expressed their opinions according to their expertise and experience. The interviews were audio-recorded with the interviewees' consent. The interviewees were a pediatrician (A), chief nurse executive from the pediatrics department of a hospital (B), hospital manager (C), medical manager from the pediatrics department of a hospital (D), architect (E), and art therapist (F).
3. Transcripts of the interviews (a total of 65,395 Chinese characters) were analyzed and decoded using the grounded theory method. Strauss and Corbin [47] stated that a data analysis process includes open, axial, and selective coding procedures. Open coding involves decomposing, examining, comparing, conceptualizing, and categorizing data. Overall, 99, 47, 37, 47, 71, and 80 concepts were extracted from interviewees A, B, C, D, E, and F, respectively. After similar concepts were combined, 292 concepts remained. Moreover, after a decoding process, 79 open codes, 25 axial codes, and 4 selective codes (i.e., emotions, space design, interpersonal interaction, and pleasant surroundings) were obtained (Supplementary Material C). The “emotional preferences” axial code in the “emotions” dimension, which was a selective code, was used as an example to explain the coding processes. As shown in Supplementary Material D, emotional preferences included open codes such as personal preferences, self-healing, and moving people emotionally. These personal preferences comprised concepts C9, E40, and E43 (C9, the ninth concept extracted from the transcript of the interview with hospital manager C, posits that art appreciation varies among people; E40 and E43, the 40th and 43rd concepts extracted from the transcript of the interview with architect E, are personalized space and differences in art preferences, resp.). Supplementary Material D details the opening coding processes for self-healing and moving people emotionally.
4. As shown in Supplementary Material E, 25 axial codes were transformed into a pilot questionnaire comprising 25 question items.
5. Pilot data were collected from May 20 to May 31, 2013. The second author of this study conducted a pilot questionnaire survey among users aged 7–18 (including inpatients, outpatients, and visitors). Before distributing the questionnaire to a participant, the researcher introduced herself and informed the participant of the purposes of the study. Because all participants had not fully experienced the entire NTUCH environment, a 5-minute, 28-second video (https://youtu.be/I4fMCC9Ca1Q) was produced to introduce the hospital environment and associated activities to the subjects. Parents assisted the younger children in completing the questionnaire. Gorsuch suggested that, for factor analysis, the ratio of the number of question items to sample size should be approximately 1 : 5 and that the total number of subjects should be more than 100 [48]. In this study, the pilot questionnaire comprised 25 questions. A total of 140 questionnaires were distributed; 128 valid questionnaires were returned. Demographics are shown in Table 1.
6. Confirmatory factor analysis and reliability analysis were conducted to determine the question items for each dimension of the final version of the questionnaire. Supplementary Material F presents a summary of the analysis process. The principal components method with varimax orthogonal rotation was used to perform factor analysis; this analysis included question items whose factor loadings were greater than .4 in the questionnaire. First, items q8 and q19 were removed according to the confirmatory factor analysis results. Subsequently, q12 and q18 were removed according to the reliability analysis results. Item q17 was next moved from the space design dimension to the interpersonal interaction dimension according to the confirmatory factor analysis results. The final version of the questionnaire comprised the 4 dimensions and 21 question items (Table 2). The four dimensions explained 59.538% of the score variance. According to the reliability analysis, the Cronbach *α* value for the four dimensions (emotions, space design, interpersonal interaction, and pleasant surroundings) and the overall value were 0.774, 0.873, 0.744, 0.8, and 0.918, respectively (Supplementary Material F). The criterion was the confirmed suitability of the questionnaire for factor analysis. Kaiser and Rice indicated that the Kaiser-Meyer-Olkin measure should not be less than 0.5. In this study, the measure was 0.875 (Supplementary Material F), indicating that it was suitable for factor analysis [49]. Internal consistency reliability was assessed using Cronbach's coefficient *α* [50]. For the questionnaire, Cronbach's *α* > 0.70 was applied as the recommended value, with *α* > 0.9 indicative of high reliability [51, 52]. As mentioned, this study exhibited construct validity and reliability.

As suggested by the results, a healing environment in a children's hospital should accommodate children's emotional preferences, be a homelike and reassuring environment that can be emotionally accepted by children, and encourage interpersonal interaction among children and between children and healthcare staff. In addition, the space design should be a visually aesthetic and child-friendly design that can provide a multifunctional and comfortable space for children. Moreover, in a children's hospital, pleasant surroundings should be created for children through both dynamic activities and static indoor and outdoor scenery.

For the formal questionnaire survey, participants were required to rate each question on a 5-point scale with anchors ranging from 1 (*strongly disagree*) to 5 (*strongly agree*). Each participant was required to provide demographic information.

### 3.2. NTUCH and Study Participants

#### 3.2.1. NTUCH

**Figure 1:**
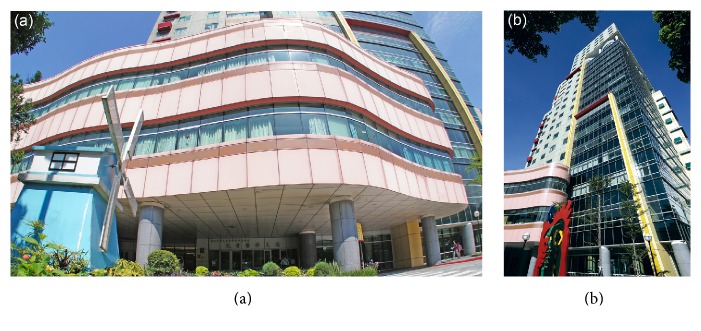
(a) Exterior of NTUCH (front view) and (b) exterior of NTUCH (side view).

**Figure 2:**
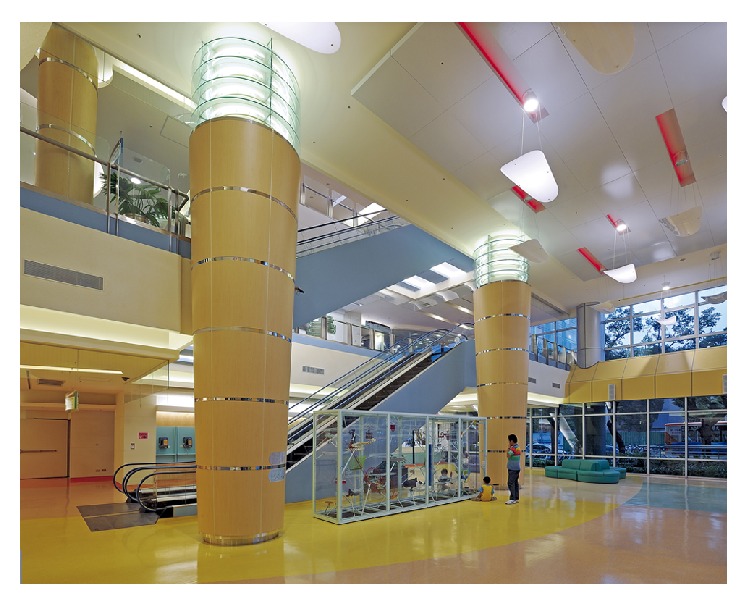
NTUCH lobby.

National Taiwan University Hospital is the oldest hospital in Taiwan, having provided healthcare services since 1895. The affiliated NTUCH was the first children's hospital in Taiwan to adopt a healing environment design. The NTUCH building has a glass curtain wall design (Figure 1), enabling a substantial amount of natural sunlight to enter the building through the windows. Colorful and uniquely shaped artworks including those suspending from the ceiling are installed in the hallways (Figure 2). The characteristics of the hospital's healing environment design are described in Supplementary Material B, and nonpharmacological intervention measures during children's medical procedures versus elements of the healing environment in the NTUCH are shown in Table 3.

#### 3.2.2. Study Participants

Children who visited NTUCH were invited to participate in this study. According to the United Nations Convention on the Rights of the Child, “children” refers to people under 18 years old. On the basis of the requirement that respondents could express themselves clearly or read, children aged 7–18 who were outpatients or inpatients or accompanied their family members to the hospital were recruited to participate in this study.

### 3.3. Data Collection

The data were collected from June 3 to June 28, 2013. Informed consent was obtained from each participant. The data collection procedure was the same as that of the pilot data sampling. The sample size *n* was determined as follows [53]:

(1) $$\begin{array}{c} n\geq {\left(\;\frac{k}{\alpha }\;\right)}^{2}p\left(\;1-p\;\right). \end{array}$$

The value of *p* is typically set at .5. In social and behavioral science research, the value of *α* (i.e., the significance level) is typically set at .05; therefore, the value of *k* is 1.96. Under these conditions, the sample size *n* should be equal to or greater than 384. In this study, the sample size was 430, and the number of valid questionnaires was 401. Hence, the return rate was 93.26%. Demographics are shown in Table 4.

### 3.4. Limitations

According to Piaget [54], children progress through four stages of cognitive development: sensorimotor (0–2 years old), preoperational (2–7 years old), concrete operational (7–11 years old), and formal operational (above 11 years old). Piaget noted that, during the preoperational stage of cognitive development, children do not yet understand concrete logic and cannot mentally manipulate information. The concrete operational stage is characterized by an appropriate use of logic, and the formal operational stage is demonstrated through the logical use of symbols related to abstract concepts. Because children's abilities to express themselves and read are limited, the participants recruited for this study were children aged 7–18. A limitation of this study was that all participants had not fully experienced the entire environment of the hospital and its associated activities. Therefore, although the participants had experienced the hospital environment, they were shown a video introducing the hospital healing environment to ensure that they understood the entire healing environment. This study aimed to identify an evidence-based method for designing and establishing a healing environment. It did not include the comparison of kids' perceptions of a healing environment with those of a regular hospital environment.

## 4. Results and Discussion

The researchers used SPSS 20 software for data analysis, and they calculated the means and standard deviations of participant perception of the healing environment according to the four dimensions and overall perception. Two-way analysis of variance (ANOVA) was performed to examine the effects of age and sex on the perception of the healing environment in each dimension and on the overall perception.

### 4.1. Descriptive Statistics for Children's Perceptions of the NTUCH Healing Environment

As shown in Table 5, the average scores of all the children's responses to the four dimensions (emotions, space design, interpersonal interaction, and pleasant surroundings) and the average overall perception score were 4.21, 4.41, 4.09, 4.17, and 4.26, respectively. The average overall perception scores of the girls and boys were 4.29 and 4.23, respectively. The trends of the average scores for the four dimensions were consistent between the boys and the girls; specifically, for both the boys and the girls, the average scores for space design were the highest, followed by those for emotions, pleasant surroundings, and interpersonal interaction. Across all age levels, the children were most satisfied with the space design dimension and least satisfied with the interpersonal interaction dimension. The trends of the average scores for the four dimensions were consistent among the children at all age levels. All the average scores for each dimension also exceeded 3.90, signifying that all the age groups positively accepted the NTUCH healing environment. Moreover, the younger age groups accepted the healing environment to a greater extent than did the older age groups; the mean perception scores for the groups aged 7–9, 10–12, 13–15, and 16–18 years were 4.39, 4.32, 4.18, and 4.10, respectively (Table 5). In all groups, boys aged 16–18 also demonstrated the lowest average scores for the four dimensions and overall perception (4.02, 3.64, 3.73, 3.87, and 3.89, resp.; Table 5).

### 4.2. Effects of Sex and Age on Children's Perception of the NTUCH Healing Environment

As shown in Table 6, the interaction effects of sex and age on emotions, space design, interpersonal interaction, pleasant surroundings, and overall perception were nonsignificant.

Regarding the emotions dimension, the main effect of sex was nonsignificant (*F* = .832, *p* > .05) and that of age was significant (*F* = 4.054, *p* = .007). Fisher's least significant difference (LSD) post hoc test revealed that the average score of children aged 7–9 for the emotions dimension was significantly higher than those of children aged 13–15 and 16–18.

For the space design dimension, the main effects of sex (*F* = 4.626, *p* = .032) and age (*F* = 3.482, *p* = .016) were significant. The average score of girls was significantly higher than that of boys. Fisher's LSD post hoc test showed that the average score of children aged 7–9 for this dimension was significantly higher than that of children aged 16–18.

Regarding the interpersonal interaction dimension, the main effect of sex was nonsignificant (*F* = 1.991, *p* > .05), whereas that of age was significant (*F* = 5.690, *p* = .001). Fisher's LSD post hoc test indicated that the average scores of children aged 7–9 and 10–12 for this dimension were significantly higher than those of children aged 13–15 and 16–18.

For the pleasant surroundings dimension, the main effect of sex was nonsignificant (*F* = 2.893, *p* > .05), whereas that of age was significant (*F* = 14.764, *p* = .000). Fisher's LSD post hoc test revealed that the average scores of children aged 7–9 and 10–12 for this dimension were significantly higher than those of children aged 13–15 and 16–18.

Concerning the overall perception dimension, the main effect of sex was nonsignificant (*F* = 3.276, *p* > .05), whereas that of age was significant (*F* = 7.034, *p* = .000). Fisher's LSD post hoc test indicated that, regarding the overall perception, the average score of children aged 7–9 was significantly higher than those of children aged 13–15 and 16–18, and that of children aged 10–12 was significantly higher than that of those aged 16–18.

### 4.3. Discussion

The statistical analysis conducted on the effect of age on children's perception of the healing environment according to the four dimensions (i.e., emotions, space design, interpersonal interaction, and pleasant surroundings) reveals that the average scores of younger children were significantly higher than those of older children. According to previous studies (Table 3), applying nonpharmacological intervention measures to manage stress, anxiety, and pain has become a widely recognized method. This type of intervention includes attention distraction strategies, such as using healing gardens [18], games [51], music [20], electronic game devices [21], clown doctors [22, 23], virtual reality [24, 25], and cartoons [26], or game-based cognitive learning tools, such as computer games and educational healing toys [27, 28]. These intervention measures target children aged 2–18. At NTUCH, most of these intervention strategies were used with children under 12 years old. Healing gardens, virtual reality, and computer games were also used with children above 12 years old (Table 3). Landreth [5] indicated that toys and games are at the center of children's lives and that using toys and games to communicate with children is a natural method that can facilitate establishing a relationship between children and healthcare staff. Children use digital toys and games that help them to pursue dominant values [55].

Because younger children typically spend considerable time playing games, nonpharmacological intervention measures can be effectively applied among them. Previous studies have also demonstrated that younger children exhibit stronger anxiety and pain responses during diagnosis and treatment processes than do older children [24, 56], signifying that more attention should be paid to younger children during healthcare processes. The results of previous studies are mainly applicable to younger children. In addition to virtual reality and computer games, other healing elements have been applied in the NTUCH healing environment (Table 3), and this may explain why the younger children accepted the healing environment to a higher degree in each of the four dimensions compared with the older children.

The sex variable significantly affected children's perception of space design, and the average score of the girls was significantly higher than that of the boys. The results are consistent with those reported by Mourshed and Zhao [15], who studied healthcare providers' perceptions toward hospital environment design factors and determined that females are more sensitive to healthcare environments than males. Moir and Jessel [57] (pp. 17-18) reported that females see larger images than males do, and this is because females have wider peripheral vision and their retina contains more cone and rod cells for receiving visual information.

According to the preceding results, children aged 7–18 positively accepted the NTUCH healing environment; in addition, they were most satisfied with space design and least satisfied with interpersonal interaction. Poor outdoor playground design reduces the frequency of children's interaction with the opposite sex [58]. NTUCH should thus improve its interpersonal interaction design to enhance healing effects. Prensky [59] reported that playing with others is fun and helps a player become involved in a community. Space design can be considered a component of hardware facilities. As presented in Supplementary Material B, game rooms (e.g., the toy room, colorful sky cave, touching republic, and youth blog), the hanging garden, family resource center, and waiting areas (e.g., fantastic journey, fantastic forest, Buddi's adventure, and animal carnival) were the healing elements that attracted children. The elements of space design were installed in the open space of NTUCH and were readily available to children in the hospital. This space design is in accordance with the suggestions proposed by Turner et al. [60] regarding a hospital's physical environment. This may be the reason why the children were most satisfied with space design.

## 5. Conclusion and Recommendation

An evidence-based healthcare design should be based on the evaluation of the perceptions of hospital care unit users [9]; this design strategy was used in the current study, and the results are as follows:

1. This study proposed qualitative and quantitative analyses and systematically summarized essential factors for designing and evaluating a healing environment in a children's hospital. Questionnaires about the design of a healing environment in a children's hospital were collected and analyzed, and related elements are summarized in this paper (Table 2 and Supplementary Material E). These elements include 4 dimensions, namely, emotions, space design, interpersonal interaction, and pleasant surroundings, and 21 elements.
2. Children aged 7–18 positively accepted the NTUCH healing environment, and they were most satisfied with space design and least satisfied with interpersonal interaction. Two-way ANOVA was performed to investigate the effects of sex and age on children's perception of the healing environment. The results indicate that the average score of girls was significantly higher than that of boys for space design, whereas the effect on the other dimensions and overall perception was nonsignificant. The main effect of age on the four dimensions and overall perception was significant; specifically, younger children accepted the healing environment to a higher degree than did older children.

This study proposes a method for evaluating the perceptions of users of children's ward healing environments, and the proposed method is applicable to children aged 7–18. Future studies should include children aged 2–6. Nevertheless, children at these ages are limited in their ability to express themselves and read; therefore, other evaluation methods should be considered. In addition, further research is necessary to examine the comparison of kids' perceptions of a healing environment with those of a regular hospital environment.

## Supplementary Material

The supplementary material a, b, c, d, e, and f are “questionnaire on the healing environment design of a children's hospital,” “the characteristics of the hospital's healing environment design,” “summary of concepts, open codes, axial codes, and selective codes,” “an example to explain the coding processes,” “open codes, axial codes, and question items for the pilot questionnaire,” “the summary of the analysis process,” respectively.

## Figures and Tables

**Table 1:** Demographic information on the pilot questionnaire subjects.

Aged 7–18	7	8	9	10	11	12	13	14	15	16	17	18	Sum
Girls	7	7	7	10	10	9	3	3	3	6	5	6	76
Boys	7	6	6	7	6	7	3	2	2	2	2	2	52
Children	14	13	13	17	16	16	6	5	5	8	7	8	128

**Table 2:** Resulting questionnaire items after grounded theory analysis versus those after factor and reliability analyses.

Dimension	Emotions	Space design	Interpersonal interaction	Pleasant surroundings
Resulting question items for the pilot questionnaire after grounded theory analysis	q1, q2, q3, q4, q5, q6	q7, q8, q9, q10, q11, q12, q13, q14, q15, q16, q17, q18	q19, q20, q21	q22, q23, q24, q25
Resulting question items according to factor analysis and reliability analysis (question items for the formal questionnaire)	q1, q2, q3, q4, q5, q6 (01, 02, 03, 04, 05, 06)	q7, q9, q10, q11, q13, q14, q15, q16 (07, 08, 09, 10, 11, 12, 13, 14)	q17, q20, q21 (15, 16, 17)	q22, q23, q24, q25 (18, 19, 20, 21)

*Note*. q8, q12, q18, and q19 were removed from the pilot questionnaire, and q17 was moved from the space design dimension to the interpersonal interaction dimension.

**Table 3:** Nonpharmacological intervention measures during children's medical procedures versus elements of the healing environment in the NTUCH.

Intervention elements	Aim	Objective	Elements of the healing environment
Healing garden [18]	Anxiety, sadness, anger, worry, fatigue, and pain	Child (2–12 years old), adolescent (13–18 years old), and adult	Hanging garden
Playful activities [19]	Anxiety	5 to 12 years old	Game room (colorful sky cave, touching republic, toy room, and youth blog), toy building bricks, craft activities, and balloons of various shapes activities
Music [20]	Anxiety and pain	2–12 years old	Regular concerts
Electronic game [21]	Child distress	3 to 7 years old	None
Clown [22, 23]	Anxiety/anxiety	3–8 years old/5–12 years old	Clown and magic shows and theatres
Virtual reality [24, 25]	Anxiety and pain/anxiety and pain	7–14 years old/6–14 years old	None
Cartoon movie [26]	Distress, restraint, and pain	4 to 6 years old	Cartoon movies shown in waiting areas
Computer game [27]	Anxiety, symptom, and emotion	10–16 years old	None
Instructional therapeutic toy [28]	Pain	3–10 years old	Seeds-of-hope hospital activities

**Table 4:** Study subjects demographic information.

Aged 7–18	7	8	9	10	11	12	13	14	15	16	17	18	Sum
Girls	18	18	12	24	22	18	20	18	15	18	18	14	215
Boys	20	18	16	22	20	22	15	14	12	10	9	8	186
Children	38	36	28	46	42	40	35	32	27	28	27	22	401

**Table 5:** Descriptive statistics for the perceptions of children toward the NTUCH healing environment.

Sex	Age (~years old)	Mean					Standard deviation					Frequency
		Emotions	Space design	Interpersonal interaction	Pleasant surroundings	Overall	Emotions	Space design	Interpersonal interaction	Pleasant surroundings	Overall	
Girls	7–9	4.35	4.51	4.23	4.44	4.41	.565	.595	.845	.695	.545	48
	10–12	4.26	4.48	4.27	4.37	4.37	.624	.545	.637	.632	.547	64
	13–15	4.08	4.38	3.89	3.92	4.14	.540	.555	.640	.552	.479	53
	16–18	4.18	4.44	4.04	4.01	4.22	.575	.561	.721	.610	.531	50
	Average	4.22	4.45	4.11	4.19	4.29	.584	.561	.720	.657	.534	215

Boys	7–9	4.31	4.49	4.22	4.35	4.37	.532	.472	.700	.708	.494	54
	10–12	4.22	4.39	4.09	4.23	4.27	.586	.540	.711	.594	.530	64
	13–15	4.24	4.41	4.07	3.98	4.23	.584	.521	.633	.654	.518	41
	16–18	3.89	4.02	3.64	3.73	3.87	.632	.738	.745	.707	.614	27
	Average	4.20	4.37	4.06	4.14	4.23	.589	.567	.715	.687	.549	186

All children	7–9	4.33	4.50	4.23	4.39	4.39	.545	.531	.768	.700	.516	102
	10–12	4.24	4.44	4.18	4.30	4.32	.603	.542	.678	.615	.538	128
	13–15	4.15	4.39	3.97	3.94	4.18	.562	.538	.639	.596	.496	94
	16–18	4.08	4.29	3.90	3.91	4.10	.608	.655	.749	.655	.583	77
	Average	4.21	4.41	4.09	4.17	4.26	.585	.564	.717	.671	.541	401

**Table 6:** Summary of two-way analysis of variance results regarding the effects of sex and age on children's perception of the NTUCH healing environment.

Dependent variable (dimension)	Source	Type III sum of squares	df	Mean square error	*F*	*p*	Eta-squared	Post hoc comparisons
Emotions	Sex	.278	1	.278	.832	.362	.002	
	Age	4.070	3	1.357	4.054^*∗*^	.007	.030	7–9 > 13–15 years old 7–9 > 16–18 years old
	Sex × age	1.976	3	.659	1.968	.118	.015	

Space design	Sex	1.436	1	1.436	4.626^*∗*^	.032	.012	Girls > boys
	Age	3.243	3	1.081	3.482^*∗*^	.016	.026	7–9 > 16–18 years old
	Sex × age	2.302	3	.767	2.472	.061	.019	

Interpersonal interaction	Sex	.984	1	.984	1.991	.159	.005	
	Age	8.437	3	2.812	5.690^*∗*^	.001	.042	7–9 > 13–15 years old 7–9 > 16–18 years old 10–12 > 13–15 years old 10–12 > 16–18 years old
	Sex × age	3.668	3	1.223	2.474	.061	.019	

Pleasant surroundings	Sex	1.183	1	1.183	2.893	.090	.007	
	Age	18.121	3	6.040	14.764^*∗*^	.000	.101	7–9 > 13–15 years old 7–9 > 16–18 years old 10–12 > 13–15 years old 10–12 > 16–18 years old
	Sex × age	1.163	3	.388	.948	.417	.007	

Overall perception	Sex	.914	1	.914	3.276	.071	.008	
	Age	5.889	3	1.963	7.034^*∗*^	.000	.051	7–9 > 13–15 years old 7–9 > 16–18 years old 10–12 > 16–18 years old
	Sex × age	2.013	3	.671	2.404	.067	.018	

^*∗*^ *p* < .05.
