# Supplementary material for: Kids' Perceptions toward Children's Ward Healing Environments: A Case Study of Taiwan University Children's Hospital
Source: J Healthc Eng. 2016 Oct 5;2016:8184653. doi: 10.1155/2016/8184653 (PMC5072350; doi:10.1155/2016/8184653)
Supplement: Supplementary file 1 — The supplementary material a, b, c, d, e, and f are “questionnaire on the healing environment design of a children's hospital,” “the characteristics of the hospital's healing environment design,” “summary of concepts, open codes, axial codes, and selective codes,” “an example to explain the coding processes,” “open codes, axial codes, and question items for the pilot questionnaire,” “the summary of the analysis process,” respectively. [file 8184653.f1.docx]

**SUPPLEMENTARY MATERIAL A. QUESTIONNAIRE ON THE HEALING ENVIRONMENT DESIGN OF A CHILDREN’S HOSPITAL**

This is a questionnaire that addresses the healing environment design of a children’s hospital. Please answer the following questions. We greatly appreciate your cooperation in completing this questionnaire.

- - 1. How do you think “a healing environment” should be defined?
    2. Regarding the construction of a healing environment in a children’s hospital, what aspects of hardware (e.g., furnishings, colors, artwork, interactive art, gardens, indoor potted plants, a view of the outdoors, lighting, and music) and software (e.g., management, the interaction between healthcare staff and children, and hospital activities) should be considered?
    3. Regarding the environment design of a children’s hospital, how can information about other healthcare institutions and building regulations be applied to health care, outpatient clinics, wards, and public activity spaces?
    4. Hospitals often use landscape design and green plants to improve their healthcare environment and quality. How do you think the design of a children’s hospital can include the elements of a natural healing environment? Please give an example.
    5. How do you think health care is related to art?
    6. How do you think public art in hospitals should be defined?
    7. What are the functions of public art in the healing environment of a children’s hospital?
    8. After open space facilities and public art are installed in a children’s hospital, what precautions should be taken to protect and maintain the facilities and public art?

**SUPPLEMENTARY MATERIAL B. THE CHARACTERISTICS OF THE HOSPITAL’S HEALING ENVIRONMENT DESIGN**

1. Waiting area: The design of the waiting area for children comprises four themes including “fantastic journey” (Figure B1a), “fantastic forest” (Figure B1b), “Buddi’s adventure” (Figure B1c), and “animal carnival” (Figure B1d). The four themes presented in the waiting area provide children with visual simulation derived from interesting decorations and prominent wall designs, which also provide children with an understanding of animals, forests, and the human body. In the waiting area, children can communicate with their own body by touching objects and listening to sounds. The interactive game space can attract children’s attention and enable children to interact with one another during the game. The room is primarily decorated with blue, green, orange, and yellow colors and was designed using nature, forests, animals, and astronomy as themes. Rich colors, interesting patterns, and a vivid atmosphere can distract children’s attention from their pain and relieve their physical and emotional stress.


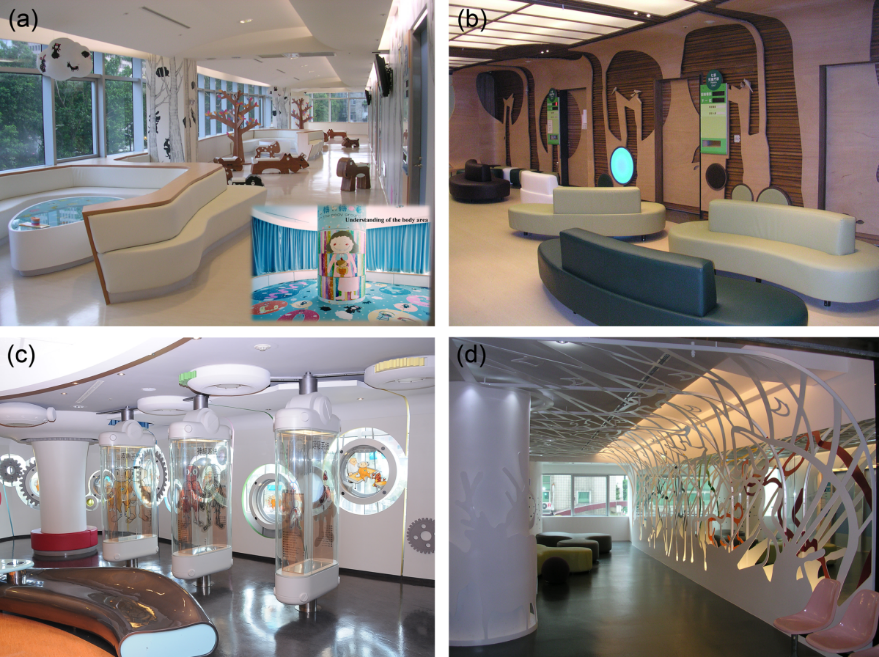


**Figure B1.** Waiting area: (a) fantastic journey, (b) fantastic forest, (c) Buddi’s adventure, (d) animal carnival.

1. Ward area: In addition to bed headboard and floor design in wards (Figure B2), game rooms with themes such as “colorful sky cave” (Figure B3a), “youth blog” (Figure B3b), “toy room” (Figure B3c), and “touching republic” (Figure B3d) were established throughout the children’s wards. A vivid, warm, and comfortable environment was created using various colors. The pleasant and relaxing environment can provide leisure for children and enhance their physical and mental health. In the “colorful sky cave” room, children can use their creativity to draw and paint. The small colorful glass cave by the door is intriguing and each cave on the wall contains a surprise. The rainbow magnetic wall exhibits children’s works and can represent a piece of collective children’s art. In the “youth blog”, which has a leisure function, notebook computers are suspended on a steel pipe. Children can sit in a donut-shaped lounge, type on the notebook computers, and connect with the world through the Internet. The simulated turf and warm sunlight inspire children’s imagination. The “toy room” resembles an oval-shaped hive. The oval-shaped design simulates a womb and provides children with a sense of security. The grid structure in the toy room creates changeable and intriguing spaces with numerous toys to attract the interest of children. The “touching republic” resembles a cave composed of ice cubes. Children can dance in the simple cave, which produces various visual and audio effects, and they can experience novel, changeable, and diverse tactile and visual stimuli.


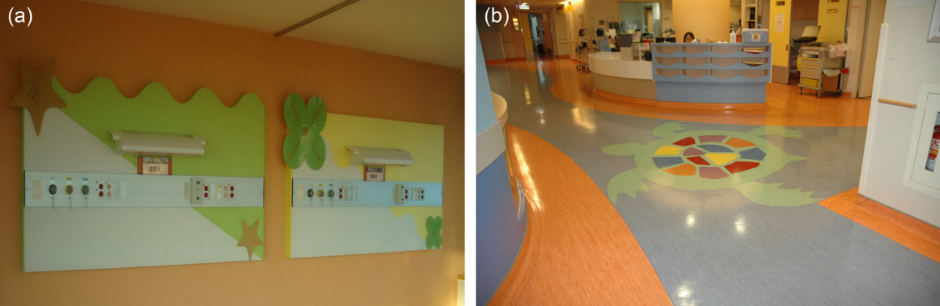


**Figure B2.** Ward area: (a) bed headboard design, (b) floor design.


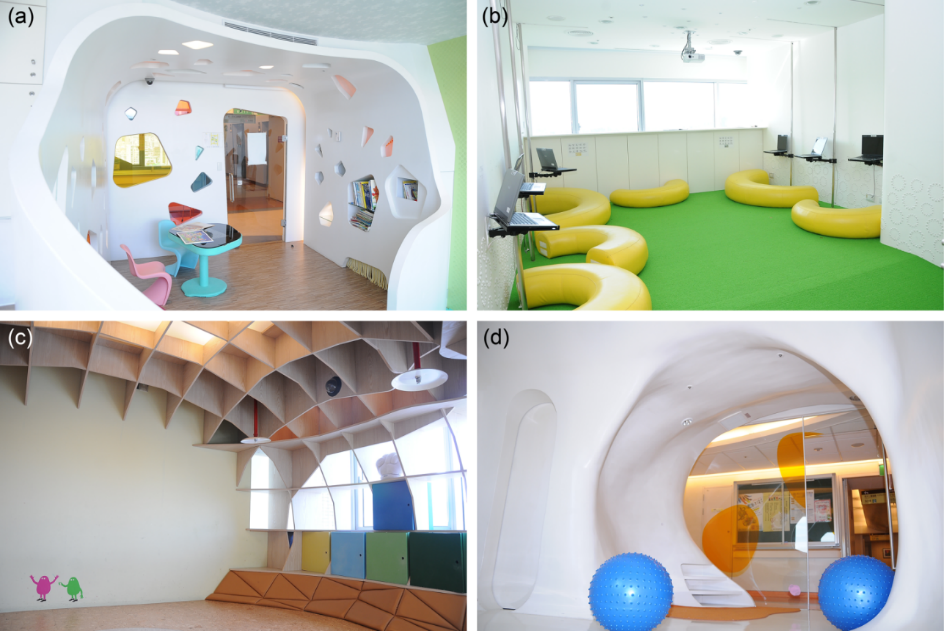


**Figure B3.** Game rooms: (a) colorful sky cave, (b) youth blog, (c) toy room, (d) touching republic.

1. Family resource center: The family resource center (Figure B4) designed using “underwater world” as the theme provides free healthcare information and educational materials, reduces anxiety in parents and children, and serves as a family library. Through reading, people who visit the hospital can relieve their emotional stress, become calm, and receive the benefits of bibliotherapy.


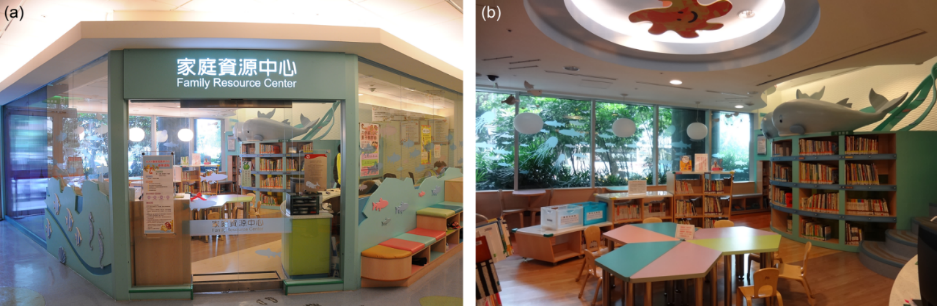


**Figure B4.** (a) The family resource center, (b) interior of the family resource center.

1. Aesthetic and educational elevator waiting area (Figure B5): People can appreciate the ecological beauty of Taiwan in the busy elevator waiting area.


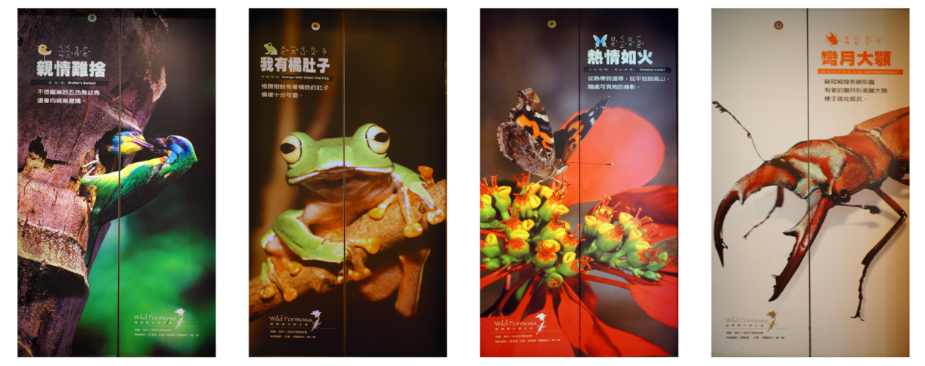


**Figure B5.** Elevator landscaping and ecological education—understanding the beauty of Taiwan.

1. Hanging garden (Figure B6): The open-air hanging garden is a lush green environment created using natural materials. A wood platform surrounded by colorful flowers provides a natural feeling. To enable children to interact with nature, the height of the flowerbeds was reduced to provide children with an opportunity to recognize various plants.

**
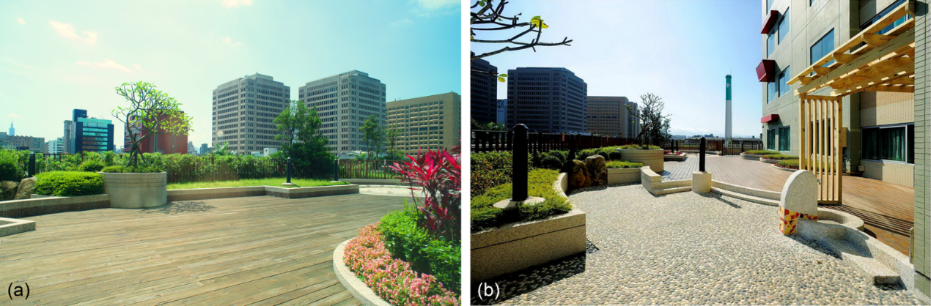
**

**Figure B6.** Hanging garden view.

1. Public artworks: The proposal for the design of the NTUCH clearly stated that the purpose of public art in the children’s hospital building was to provide children with a joyful and colorful amusement park. Sixteen Taiwanese and foreign artists were invited to create 17 works of art to be installed on various floors of the children’s hospital following the concept of a “health forest.” The materials used included steel, stone, wood, copper, ceramics, glass, blankets, cloth, acrylic, and digital media. For example, in the area outside the entrance to the first floor, two outdoor sculptures were installed (Figure B7). A dynamic mechanical piece of art, the funny rolling ball unit (Figure B8), was installed in the lobby on the first floor. In addition, pieces such as “I am a Drug Store” (Figure B9a), “Happily Growing” (Figure B9b), and “Tree of Hope” (Figure B9c) were installed in the ophthalmology waiting area on the first floor, at the west nursing station in the ward area on the 12^th^ floor, and at the east nursing station in the ward area on the 14^th^ floor, respectively. According to the function and atmosphere of each floor, diverse and colorful works of art were designed and installed to reduce outpatient and inpatient children’s fear and anxiety. In addition, the public works of art installed on each floor can be used to distinguish the floor from other floors.


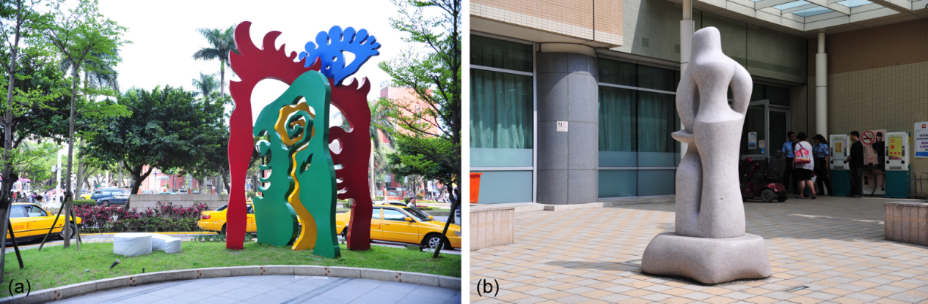


**Figure B7.** Outdoor sculptures: (a) colorful castle of love, (b) dancing sculpture.

**
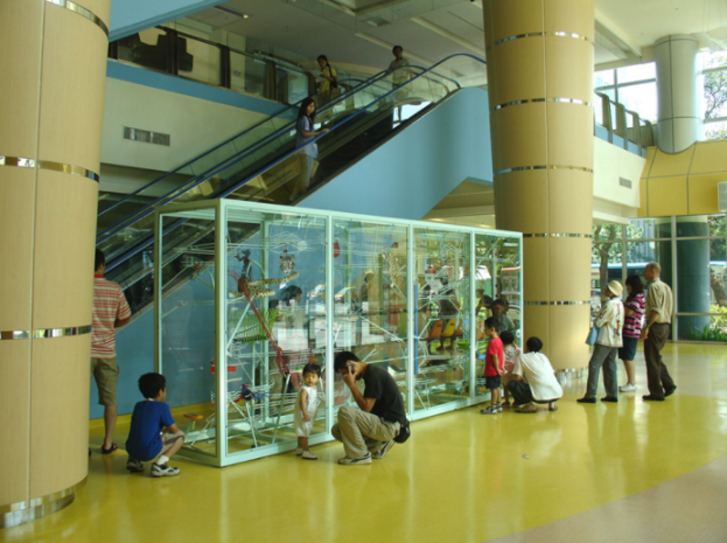
**

**Figure B8.** Funny rolling ball unit.

**
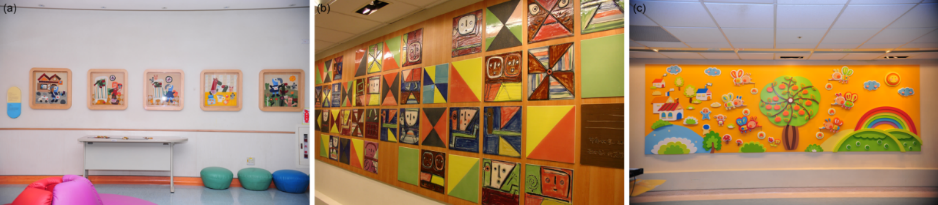
**

**Figure B9.** (a) I am a drug store, (b) happily growing, (c) tree of hope.

1. Joyful and healing activities: Activities organized by the NTUCH for children and their family members can be categorized into six types: (1) seeds-of-hope hospital activities (Figure B10a); (2) ward activities (Figure B10b); (3) periodic literary activities (Figure B10c); (4) children’s storytelling and reading activities (Figure B10d); (5) festival activities (Figure B10e); and (6) enterprises giving back to society (Figure B10f). Seeds-of-hope hospital activities originated from Teddy Bear Hospital (TBH). TBH is an international project and its purpose is to reduce children’s anxiety toward healthcare environments and procedures. Regarding ward activities, because children inpatients’ ability to move is limited and they cannot participate in public events, bedside activities, craft activities, and interactive puppet shows can provide joy for children. Regular literary activities such as concerts, children’s shows, and do-it-yourself activities can make child patients feel as if they are in a playground. Storytelling activities can distract children’s attention from their illness and a happy story containing positive characters can create a positive mood in children. Regarding festival activities, the hospital designs diverse programs for major festivals such as magic shows, clown acts, and caroling with Santa Claus to express their care for child patients and their family members. Regarding enterprises giving back to society, enterprises organize singing and dancing activities to assist children in forgetting about their discomfort.


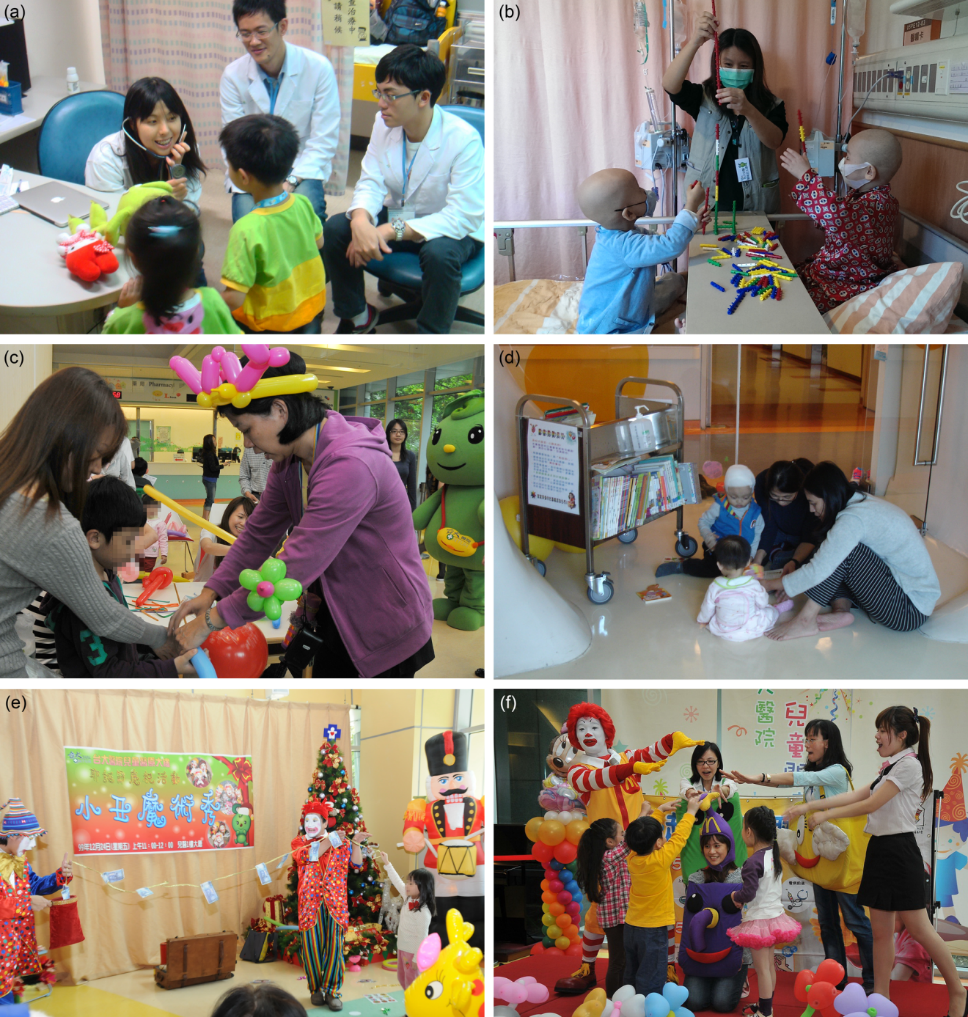


**Figure B10.** Joyful and healing activities (a) seeds-of-hope hospital activities, (b) bedside caring activities—Playing with toy building bricks, (c) creating various balloon shapes activities, (d) book cart—family reading activities, (e) clown and magic shows, (f) Ronald McDonald House Charities.

**SUPPLEMENTARY MATERIAL C. SUMMARY OF CONCEPTS, OPEN CODES, AXIAL CODES, AND SELECTIVE CODES**

| **Dimension** | **Axial code** | **Open code** | **Concept** |
| --- | --- | --- | --- |
| Emotions | Emotional preferences | Personal preferences | E40, E43, C9 |
|  |  | Self-healing | E2, E3, E12, E20, E35 |
|  |  | Emotionally moving people | A86, E46, E47, F69 |
|  | Emotional identification | Affecting emotions | A6, E5, E18, E26, F11 |
|  |  | Religious beliefs | A42, A43, A41 |
|  |  | Changing thoughts | E22 |
|  |  | Receiving messages | E23 |
|  | Identification with artwork | Interaction with art | A28, C14, F40, F55 |
|  |  | Layout perception | A27, A55, A68, C27, E4, E24, E52, E53, E57, E59 |
|  | Home-like comfort | Personal privacy | F1, E42 |
|  |  | Home-like atmosphere | E41 |
|  | Children’s requirements | Child psychology | A8, B9, F3, F7, F70, F71, B2 |
|  |  | Stress relief | B11, B13, B21, B23, E11 |
|  | Secure design | Safety | A64, D35, D44, D47, E25, D71, F5, F17, F47 |
|  |  | Infection control | A63, B3, B43, F18, F21 |
|  |  | Barrier-free environment | E10 |
| Space design | Design and regulations | Government regulations | D13, E17, E63, E65 |
|  |  | Ward design | B7, D2 |
|  | Educational function | Story implications | A80 |
|  |  | Life experiences | F67, F68 |
|  |  | Nature education | B45 |
|  | Functional design | Functionality | E30, F72 |
|  |  | Spatial mobility | A26 |
|  |  | Publicity | E39 |
|  |  | Diversity | A10, A61, A65, C28, F16 |
|  |  | Theme design | B17, D29, F34, F35, F51, A76 |
|  |  | Integrated design | E33, A96 |
|  |  | Local elements | A15, A16, A19, A30, A31, C13 |
|  | Age level | Suitable design for a certain age level | A12, D9 |
|  |  | Suitable design for users | A67, B31, C3, C4, F33, A41, F44 |
|  |  | Popularization | D17, E15, F23, F30, F41, F42, F43 |
|  | Children’s elements | Suitable design for children | A13, A44, A79, B25, B27, C8, D1, D14, D31, D39, E44, F36, C5, E16, F14 |
|  |  | Cartoonization | B28, B40, C26 |
|  |  | Fun | A18, A24, A39, A54, A71, A75, A85, A94, B19, B32, B39, B41, C11, C12, C22, C25, D18, D28, E54, F19, F26, F28, F29, F50 |
|  |  | Creativity | E70 |
|  |  | Closeness | A66, C10, C19, E38, F20, F59 |
|  |  | Children’s elements | D32 |
|  |  | Colorfulness | A20, A22, A23, A33, A34, A78, B14, B26, C23, D3, D30, F64 |
|  | Facility maintenance | Facility maintenance | A92, A97, A98, A99, B37, C2, C15, C30, C31, C32, C33, C34, D41, D43, D45, D46, E58, E60, F24, F56, F57, F61 |
|  | Corporate costs | Durability | A90, A91, B36, D40, D42 |
|  |  | Labor costs | F6 |
|  | Design by professional teams | Curatorial team | C17 |
|  |  | Team communication | A35, C29, D4, D20 |
|  |  | Team design | C7 |
|  | Relaxing landscape | Ease | A38 |
|  |  | Healing garden | A14, A37, A83, A89 |
|  |  | View of the outdoors | B8, D23, D24, D27, D36, D37, F46 |
|  |  | Natural elements | A48, A73, F52, E51 |
|  |  | Comfortable environment | A36, D22, E7, E19, E32 |
|  | Art design | Integrative art | E1, E31, E34, F32, C6 |
|  |  | Art therapy | A46, A9, A59, A84, F8, F10, B12 |
|  | Attention distraction | Attracting attention | B18, B22, B33, B34, B35, C20, E13, F31, F49, F53, F60, F63 |
|  |  | Novelty | A69, A70, F65 |
|  |  | Interaction | F22, F58, F62, A29, A81, A95 |
|  |  | Richness | A1, B24, B38 |
|  | Indicator guidance | Landmarks | A17, A25, E37 |
|  |  | Hospital integration | A4, A51, B16, C1, D19, E15, E42, F13 |
|  |  | Indicator recognition | A77, C24, E36, E56, F25, F27, F66, F75 |
|  |  | Unified style | A32, A72 |
|  |  | Understanding of the environment | A11 |
| Interpersonal interaction | Emotion communication | Interaction with people | A87, A93, B46, F48 |
|  |  | Interpersonal relationships | E8, E64 |
|  |  | Relationship between patients and healthcare staff | F4 |
|  |  | Volunteer services | B5, E62 |
|  | Services and communication | Professional services | C36 |
|  |  | Holistic care | F9, F12 |
|  |  | Dynamic medical consulting | A2 |
|  |  | Service targets | D21, D38 |
|  | Playful atmosphere | Playful atmosphere | B4, B47, C18, F77, B6 |
| Pleasant surroundings | Participation in activities | Participating in activities | F38 |
|  |  | Participating in art activities | E66, E68, E78 |
|  | Arts and culture | Arts and cultural activities | A47, D7, D8, F37 |
|  |  | Charity activities | D10, D11, D12, E61 |
|  | Healing activities | Art creation | A53, C16, C37, E69, C21, F80, D6, F2, F79 |
|  |  | Game activities | E9, B10, E67, F39 |
|  |  | Animal doctors | B42 |
|  | Natural positivity | Natural atmosphere | D33, D34, E48, E49, F45, F54 |
|  |  | Positive atmosphere | A7, A21, A40, A45, A50, A52, A56, A58, A60, A62, A74, B20, D5, D15, D16, D25, E27, E28, E29, E45, E50, F76, A82, A88, B1, B15, B29, B30, B44, C35, E6, E14, E21, E55, F73, F74 |
|  |  | Art influence | A5, A57, D26 |

**SUPPLEMENTARY MATERIAL D. AN EXAMPLE TO EXPLAIN THE CODING PROCESSES**

| **Axial code** | **Open code** | **Concept** | **Opinions** |
| --- | --- | --- | --- |
| Emotional preferences | Personal preferences | C9: Art appreciation varies among people | From my perspective, public art displayed in the hospital can be divided into two components: the public and the art. The art should be discussed from an art appreciation perspective. Various people appraise the same painting differently. |
|  |  | E40: Personalized space | I have read a report that in an American hospital, when a patient is admitted to the hospital, a trolley will come to the patient and the patient can choose a painting from the trolley to hang in his or her ward. Isn’t that wonderful? Like choosing a gift, each patient can choose a painting he or she likes to make his or her space different from that of others. |
|  |  | E43: Differences in art preferences | People have differing taste in art. Certain people like realistic paintings and other people like abstract paintings. |
|  | Self-healing | E2: Physical self-healing | In the past, obtaining the most effective medicines was the trend. “As long as I give you the best medicine, you will recover.” Subsequently, visiting the most effective doctor was the trend. Presently, an increasing number of people believe that the human body can heal itself. |
|  |  | E3: Autologous recovery process | Several cancer patients had a cold that lasted 3 days and other cancer patients had a cold that lasted 7 days. A doctor may use various methods to prevent conditions that negatively affect patients’ health and improve beneficial processes such as autoimmunity. I believe health care is a process. |
|  |  | E12: Being alone | Speaking for myself, I started to learn piano in my adulthood. I enjoy playing piano. Playing piano is a great experience. By playing piano, I can observe my own condition. Occasionally, I feel irritation, enjoyment, boredom, or fulfillment when I play piano. Playing piano is extremely enjoyable and being with myself is great. |
|  |  | E20: Being happy when alone | Presently, I often want to engage in exercise. Therefore, I went for a walk while listening to music from my cell phone. I felt so happy. |
|  |  | E35: Self-experienced Five Senses Garden | The Five Senses Garden is for people with disabilities. In the Five Senses Garden, people can use their hands to touch plants and feel the ground under their feet. In addition, people can plant flowers. It is a very comfortable place. |
|  | Moving people emotionally | A86: Spiritual healing design | The game room on the second floor has a special design that enables people to talk about life, aging, sickness, and death in a meaningful way. When talking about death, people can imagine the ceiling as a doorway to another world where their deceased grandparents live. People who have experienced challenges may be emotionally moved or develop a pleasant mood through the use of these methods. This world is changeable; therefore, observing the world from various perspectives can facilitate healing although healing is not the only goal. |
|  |  | E46: Positive experiences that are an inspiration to people | I believe that people who stay in a hospital are not physically or mentally healthy. By recalling a pleasant experience, a person can be inspired. |
|  |  | E47: Guidance from positive experiences | A previous positive experience may provide people with inspiration to pursue a new direction in their lives. I believe that art in a hospital should also have this function. |
|  |  | F69: Association with previous experiences | Holistic care is the current trend in health care. Hospitalization experiences are also life experiences. These topics can be associated with life experiences and people can learn from these experiences. This is an association with previous experiences. |

**SUPPLEMENTARY MATERIAL E. OPEN CODES, AXIAL CODES, AND QUESTION ITEMS FOR THE PILOT QUESTIONNAIRE**

| **Selective code** | **Axial code** | **Question number** | **Question items** |
| --- | --- | --- | --- |
| Emotions | Emotional preferences | q1 | I like to visit the NTUCH despite feeling unwell. |
|  | Emotional acceptance | q2 | Doctors and nurses are nice here; the environment is comfortable and fun; I am not afraid to visit the NTUCH. |
|  | Identification with artwork | q3 | I like the art designed for children displayed at the NTUCH. |
|  | Home-like comfort | q4 | The new environment in the NTUCH is comfortable like home; I become accustomed to it quickly. |
|  | Children’s emotional requirements | q5 | When I am at the NTUCH, doctors and nurses always help me and encourage me. |
|  | Secure design | q6 | The hospital environment makes me feel safe and secure. |
| Space design | Design and regulations | q7 | The design and regulations of the NTUCH are appropriate. The style of the hospital is lively and fun; I enjoy it. |
|  | Educational function | q8 | I can learn about numerous subjects in the hospital (e.g., understanding animals and plants, natural science, and the human body). |
|  | Functional design | q9 | It is great that the hospital provides diverse themes, materials, and functions. |
|  | Age level | q10 | The design of the NTUCH is suitable for children. |
|  | Children’s elements | q11 | I believe that the design of the NTUCH is interesting and fun. |
|  | Facility maintenance | q12 | The hospital doesn’t contain broken facilities that cannot be used. |
|  | Corporate costs | q13 | I believe that the hospital strives to beautify the environment and install numerous works of public art. |
|  | Design by professional teams | q14 | It is great that the NTUCH has been carefully designed. |
|  | Relaxing landscape | q15 | Several natural and interesting landscapes around the NTUCH make me feel relaxed. |
|  | Art design | q16 | The artistic atmosphere of the NTUCH makes me feel comfortable and energetic. |
|  | Attention distraction | q17 | The NTUCH is appealing and makes me forget about stress and discomfort. |
|  | Indicator guidance | q18 | The design of the NTUCH helps me familiarize myself with the environment and, therefore, I do not become lost. |
| Interpersonal interaction | Emotion communication | q19 | I tell other people how I feel. |
|  | Services and communication | q20 | I believe that doctors and nurses are happy to explain my illness to me and listen to me. |
|  | Playful atmosphere | q21 | At the NTUCH, I feel like I am in a playground, and this makes me forget about illness and discomfort. |
| Pleasant surroundings | Participation in activities | q22 | I like to participate in children’s activities At the NTUCH. |
|  | Arts and culture | q23 | Arts and cultural activities held by the NTUCH are interesting. |
|  | Healing activities | q24 | I feel relaxed when participating in creative activities held by the NTUCH. |
|  | Natural positivity | q25 | I feel comfortable when seeing plants, landscape views, pictures, and colors in the hospital environment. |

**SUPPLEMENTARY MATERIAL F. THE SUMMARY OF THE ANALYSIS PROCESS**

| **Steps** | **Description** | **Number of question items/(Number of the question being removed)** |
| --- | --- | --- |
| 1. Factor analysis | Kaiser-Meyer-Olkin (KMO) = .862;  Bartlett’s test of sphericity: *p* = .000;  Four dimensions explained 53.547% of the variance;  The factor loadings of q8 and q19 were less than .4;  The factor loadings of other questions were greater than .4. | 23/(q8, q19) |
| 2. Reliability analysis | Cronbach's alpha = .916 for the questionnaire;  After q12 and q18 were removed, Cronbach's alpha = .918. | 21/(q8, q19, q12, q18) |
| 3. Factor analysis | KMO = .875;  Bartlett’s test of sphericity: *p* = .000;  Four dimensions explained 59.538% of the variance;  The factor loadings of all question items were greater than .4. | 21/(q8, q19, q12, q18) |
| 4. Reliability analysis | Cronbach's alpha = .918 for the questionnaire | 21/(q8, q19, q12, q18) |
